# Chagas disease challenge - New techniques for diagnosis and treatment address to control in endemic areas

**DOI:** 10.1590/0074-02760210033chgsb

**Published:** 2022-05-16

**Authors:** José Ricardo Pio Marins

**Affiliations:** 1 Secretaria Municipal de Saúde, Sorocaba, SP, Brasil

The discussion of the control of Chagas disease (CD) is a necessity, which is increasingly current and on the agenda of world public health. This disease has many facets, remaining in endemic countries and appearing in non-endemic countries, given the persistence of old ways of living of the countryman and the current, highly technological living, typical of the modern world. Large migratory movements, social upheavals, inequalities and advances in medicine with the use of blood products, the performance of organ or tissue transplants, together with rapid means of transport, has facilitated the maintenance of the dissemination of this trypanosomiasis globally.

Estimates of the number of people with CD at the end of the last decade pointed to the presence of millions affected in South America, Central America and Mexico. The forms of transmission, in several countries, were and are still, mostly vectorial, surprisingly congenital, in addition to the recently recognised oral transmission. The number of patients with chronic CD is very high, because they reflect the sum of cases from several previous years and current ones, considering that this disease has a long and chronic evolution.

At the same time, migratory pressure remains between poor and rich countries. This human movement has led a significant number of people infected by *Trypanosoma cruzi* to the United States, Europe, Australia and some Asian countries, with the spread of the infection in these regions being mainly due to the use of blood, blood products and transplants.

In addition to vectorial transmission, congenital, that occurs in endemic countries, transmission through blood and blood products, and by transplants is also observed, demonstrating the contrasts existing in these countries, that is, in addition to the old forms of transmission, more recent forms have appeared. Given these more complex forms of transmission, there are thousands of new cases of CD in several of these regions, with active disease and consequently a significant number of deaths resulting from trypanosomiasis.

The second most important cause of transmission in general, which is the main cause in non-endemic countries, is mother-to-child infection, called congenital CD. The distribution of this form of transmission is wide and remains high due to the still large number of infected or sick women of childbearing age.

Certainly the transmission by blood and blood products is a risk factor in endemic countries and therefore all blood products should be tested for CD. In non-endemic countries, screening policy varies, with some doing good-quality screening tests and others centring screening in people from endemic areas or those who have visited endemic areas.

Finally, all transplant cases need to be conducted rigorously both for the diagnosis of donor infection, drug prophylaxis in people at risk, the in-depth investigation of infection in recipients and treated donors and recipients, and whether or not there were successful therapeutic responses.

But the central point of this discussion needs to have another focus. As the article points out, there has been a large progression in diagnosis, currently with serological tests, with high sensitivity and specificity, which allow for a good diagnosis. There are also quantitative polymerase chain reaction (PCR) and mRNA PCR tests, both complex molecular biology techniques, also with excellent predictive values. Laboratory tests have been developed for the identification of trypanosome strains, which allow us to associate those strains more associated with the different forms of clinical evolution of the disease, including those at higher risk of congenital transmission.

This extensive presentation of the epidemiological situation above reveals a paradox. All the technology available, especially in rich countries, which has infected immigrants, but in small numbers when compared to endemic countries, is worrisome, given the objective of not affecting non-endemic countries. Technologies which, by the way, are very well received, but are of difficult access by endemic countries.

The contrast of this situation lies in the lack of the development of new technologies that can be used in the field, in order to eliminate the transmission vector of CD, since the endemic disease still remains. We need to create tests that can be used in the field, such as rapid tests already in use for HIV, Hepatitis, Syphilis and a multitude of other non-infectious diseases, enabling an easy diagnosis, without requiring large laboratories and thus allowing early treatment of large numbers of newly infected individuals, thus redirecting interventions.

The search for drugs or other prevention technologies, such as the use of antiretrovirals used in pre-exposure, is an example to be copied, especially for regions where environmental interventions and access to at-risk populations are still difficult.

Why do we not discuss the need to mobilise health services for the annual screening of large at-risk populations in endemic countries, why is the treatment of millions of patients not established as a priority, seeking to use the most modern interventions, but adapted to environments without major health structures?

Therapeutics visibly develop due to the social pressure that a disease constitutes to society; this statement is easily identifiable in the development of drugs against H1N1 infection, acquired immunodeficiency syndrome (AIDS) infection, more recently with Coronavirus disease 2019 (COVID-19), among many other infectious diseases or not. Of course all this technology developed for the coping of these pandemics or disease is highly valued, however, one must ask simultaneously, what technological advances have occurred to eliminate the secular CD, which drugs have emerged to cure different forms of the disease and transmission through the placental barrier and, finally, what technology has emerged to control the vectors, that allows the non-infection of these insects, in order to break the natural transmission cycle?

Recently, the World Health Organization (WHO) elected a World Chagas Disease Day, an initiative of great value, but which needs to have the goal of breaking the cycle of endemicity, needs to provide visibility to the indignation that humanity must feel with the thousands of agonising deaths annually of men, women and children, who have access to almost no benefit of new technologies and worse, not even to the oldest treatments.

The importance of new diagnostic methods, new therapies, new uses of organs at risk of infection, in short, of all that science and BIGFARMA have been developing for the management of *T. cruzi* infection is not questioned, however, we expect that the call of the reality of millions of lives also directs efforts to save them. This is the main challenge of all development.^1^
